# Enhancing regenerative medicine: the crucial role of stem cell therapy

**DOI:** 10.3389/fnins.2024.1269577

**Published:** 2024-02-08

**Authors:** Jipeng Wang, Gang Deng, Shuyi Wang, Shuang Li, Peng Song, Kun Lin, Xiaoxiang Xu, Zuhong He

**Affiliations:** ^1^Department of Gastrointestinal Surgery, Zhongnan Hospital of Wuhan University, Wuhan, China; ^2^Department of Neurology, Tongji Hospital, Tongji Medical College, Huazhong University of Science and Technology, Wuhan, China; ^3^Department of Otorhinolaryngology-Head and Neck Surgery, Zhongnan Hospital of Wuhan University, Wuhan, China

**Keywords:** stem cell therapy, secretome, regenerative medicine, mesenchymal stromal cell, regeneration

## Abstract

Stem cells offer new therapeutic avenues for the repair and replacement of damaged tissues and organs owing to their self-renewal and multipotent differentiation capabilities. In this paper, we conduct a systematic review of the characteristics of various types of stem cells and offer insights into their potential applications in both cellular and cell-free therapies. In addition, we provide a comprehensive summary of the technical routes of stem cell therapy and discuss in detail current challenges, including safety issues and differentiation control. Although some issues remain, stem cell therapy demonstrates excellent potential in the field of regenerative medicine and provides novel tactics and methodologies for managing a wider spectrum of illnesses and traumas.

## Introduction

**Figure 1:**
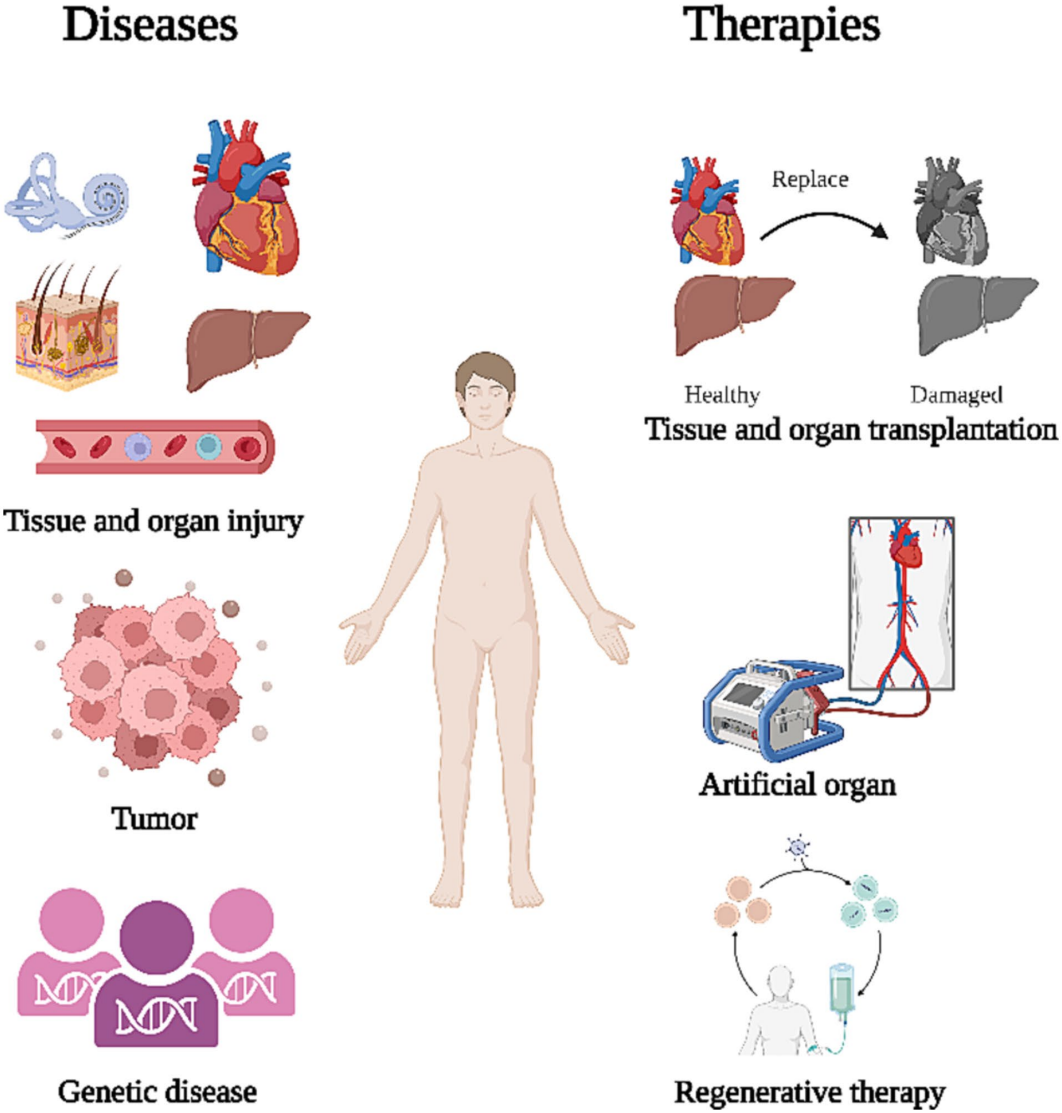
Treatment after organ dysfunction. The primary causes of organ dysfunction include tissue and organ damage, tumors, and congenital genetic diseases. To restore corresponding functions, current treatments primarily focus on organ transplantation, artificial organ substitution, and regeneration of organs.

Organ damage and degenerative diseases are caused by cell death through ageing or loss of function and can seriously affect people’s lives. Examples of such conditions include degenerative diseases like Parkinson’s disease, Alzheimer’s disease, cirrhosis of the liver, and hearing loss, as well as injurious diseases such as myocardial infarction and skin burns. Organs such as the liver have high regenerative capacity and can regenerate sufficiently to maintain functional stability under certain circumstances (Michalopoulos and Bhushan, 2021). Mouse liver has demonstrated robust regeneration that supports liver function after partial hepatectomy (Zhang et al., 2021; Duan et al., 2022; Fan et al., 2022). Unfortunately, most tissues and organs do not have such regenerative capacity and cannot repair themselves after injury, eventually leading to loss of function. An example of this would be the hair cells in the cochlea, which do not regenerate once they are damaged, resulting in irreversible hearing loss (Warchol et al., 1993). These patients will require a cochlear implant, whereby an electronic device containing an array of electrodes and a receiver is surgically implanted into the patient’s inner ear to directly stimulate the auditory nerve and recover some of the patient’s hearing (Lenarz, 2017; Carlson, 2020; Weltin et al., 2022). Similarly, patients with damaged heart valves will require replacement with artificial valves made of metal or biological material in order to maintain heart function (Singh et al., 2019; Hofferberth et al., 2020; Dreyfus et al., 2022; Figure 1).

Artificial organ replacement is complex and patients can develop infections or immune rejection after transplantation (Ko et al., 2016; Bakir et al., 2022; Crespo-Leiro et al., 2022). Some inflammatory reactions, such as infectious endocarditis, can be fatal (Berisha et al., 2022). Therefore, there is a need for an immunogenically weak treatment that can effectively repair damaged tissues and organs in patients, aiming to minimize the occurrence of adverse events. Stem cells (SCs), which have great potential to differentiate into a variety of cells and can proliferate indefinitely (Jin, 2017). SCs can be induced to differentiate into specific cell or tissue types *in vitro* before transplanting into the patient to replace degenerated or necrotic cells (Czajkowski et al., 2019; Selvaraj et al., 2019; Gholamigeravand et al., 2021; Melton, 2021). In addition, SCs can secrete anti-inflammatory factors, cytokines, and exosomes to suppress the inflammatory response and improve the microenvironment of the damaged area, ultimately regulating cell proliferation and differentiation (Vakhshiteh et al., 2019; Veneruso et al., 2019; Lv et al., 2021). This review provides a comprehensive overview of the mechanistic studies and clinical applications of stem cell therapy, while also pointing out pertinent issues in the field.

## Classification of SCs

SCs are cells with multi-directional differentiation potential while retaining the ability to replicate and renew themselves. They can be classified according to the extent of their differentiation capability (Table 1).

Totipotent stem cells (TSCs) are a type of stem cell with the remarkable ability to differentiate into any cell type within an organism, including the placental cells necessary for embryonic development (Malik and Wang, 2022). TSCs exist at the earliest stages of embryonic development, typically at the zygote stage after fertilization when a sperm cell fertilizes an egg cell. At this point, the zygote is formed, which possesses the potential to develop into a complete organism (Baumann, 2017). TSCs often have a number of unique molecular features, including lower DNA methylation (Smith et al., 2014) and activation of endogenous retroviral components (ERVs) (Hurst and Magiorkinis, 2017). The TSC state can be induced by several methods. A mixture of the GSK inhibitor 1-azakenpaullone, the retinoic acid analogue TTNPB, and the kinase blocker WS6 can induce mouse embryonic stem cells (ESCs) to exhibit a phenotype similar to that of TSCs at the fertilized egg and two-cell stages (Hu et al., 2023). Furthermore, heterochromatin remodeling has also been demonstrated to help establish allozygous-specific H3K4me3 structural domains, thus effectively facilitating the transformation of ESCs from pluripotency to allozygosity (Yang et al., 2022). The unique ability of TSCs to differentiate into whole organisms is of great interest to developmental biology and regenerative medicine research. Still, there are specific ethical issues associated with their use and study (Takahashi and Yamanaka, 2006).

Pluripotent stem cells (PSCs) are known for their exceptional ability to differentiate into various specialized cell types across all three germ layers: ectoderm, endoderm, and mesoderm. Specifically, PSCs exhibit a significant capacity for differentiation into ectodermal derivatives (Yilmaz and Benvenisty, 2019). This is evidenced by their ability to generate neurons, glial cells, neural crest cells, and other cell types originating from the ectoderm (Yang et al., 2022). They are typically derived from ESCs at the blastocyst stage (Varzideh et al., 2023), or induced pluripotent stem cells (iPSCs) by reprogramming adult cells with four transcription factors: *Oct4*, *Sox2*, *Klf4*, and *c-Myc* (Takahashi and Yamanaka, 2006). The most significant advantage of iPSCs is that they are derived from differentiated somatic cells, providing the advantages of SCs while significantly avoiding the ethical issues associated with TSCs and ESCs. The indefinite self-renewal capacity of iPSCs in culture allows the generation of an almost unlimited supply of specialized cells, offering a great potential for the study of early human development, disease modeling and regenerative therapies (Chandy et al., 2022; Cho et al., 2022; Varzideh et al., 2023).

Adult stem cells (ASCs), categorized as multipotent stem cells, demonstrate a more restricted capacity for differentiation compared to pluripotent stem cells. These cells reside in various tissues and organs throughout the body, playing a role in maintaining, repairing, and regenerating tissues within their specific microenvironments (Prentice, 2019). Unlike pluripotent stem cells, which possess a broader potential to differentiate into diverse cell types from multiple germ layers, ASCs are more constrained in their differentiation scope. They typically generate cell types specific to their tissue or organ of origin and are more specialized than pluripotent stem cells. Consequently, ASCs can only generate specific cell lineages corresponding to the exact tissue of their origin, differing from the broader differentiation potential exhibited by their pluripotent stem cell counterparts (Barker et al., 2010). The most common ASCs include hematopoietic stem cells (HSCs; responsible for the production of blood cells in the bone marrow) (Cho et al., 2022), mesenchymal stem cells (MSCs; differentiate into fat, cartilage, and bone cells in various tissues) (Wang et al., 2023), and neural stem cells (NSCs; differentiate into neurons, astrocytes, and oligodendrocytes of the nervous system) (Zholudeva et al., 2021). ASCs are characterized by their relative abundance in adult tissues, their ability to regulate the microenvironment by secreting specific signaling molecules, and their ease of isolation (Ma et al., 2014; Zholudeva et al., 2021). They are, therefore, of great value for tissue and organ repair and cancer therapy (Barker et al., 2010; Liu et al., 2023; Wang et al., 2023).

Unipotent stem cells (USCs) constitute a specialized subset among stem cells, distinguished by their notably restricted differentiation potential. In contrast to pluripotent or multipotent stem cells, which are capable of generating a variety of cell types, USCs are dedicated solely to generating a single specific cell type (Lilja et al., 2018). These cells predominantly reside in specific tissues or organs, fulfilling a crucial function in sustaining, repairing, and rejuvenating the particular tissue they inhabit (Thomson et al., 1998). USCs are commonly found in adult tissues and are able to continuously replenish particular cell populations that are consumed, playing a vital role in tissue maintenance and repair. Examples of USCs include basal cells in the skin (Lin and Lu, 2021) and satellite cells in the skeletal muscle (Mierzejewski et al., 2020). The potential of USCs in the treatment of diseases is limited by their single mode of differentiation.

**Table 1:** Classification of stem cells.

Classification	Differentiation capacity	Examples
Totipotent stem cells	The most strongest differentiation potential, able to develop into complete individuals	Early embryonic stages of the fertilized egg and two-cell stage
Pluripotent stem cells	Second only to totipotent stem cells, capable of differentiating into most cells	Embryonic stem cells and induced pluripotent stem cells
Adult stem cells	Limited differentiation potential, able to differentiate into multiple cell types in a specific tissue or organ	Hematopoietic stem cells, mesenchymal stem cells, and neural stem cells
Unipotent stem cells	The weakest differentiation capacity, capable of differentiating into only one specific cell type in its tissue of origin	Skin basal cells and skeletal muscle satellite cells

## The technical route to SC therapy

To employ SCs for therapy, it is essential to first consider the source of the SCs. Considering their differentiation capacity and ethical issues, the most widely used SCs for treatment are currently PSCs and ASCs. ESCs are typically derived from the inner cell mass of a blastocyst (Bacakova et al., 2018). iPSCs are generated as described above and ASCs are derived from a variety of adult tissues, including adipose tissue, bone marrow, neural tissue, blood, skeletal muscle, etc., which provide convenient cell sources (Bacakova et al., 2018).

**Figure 2:**
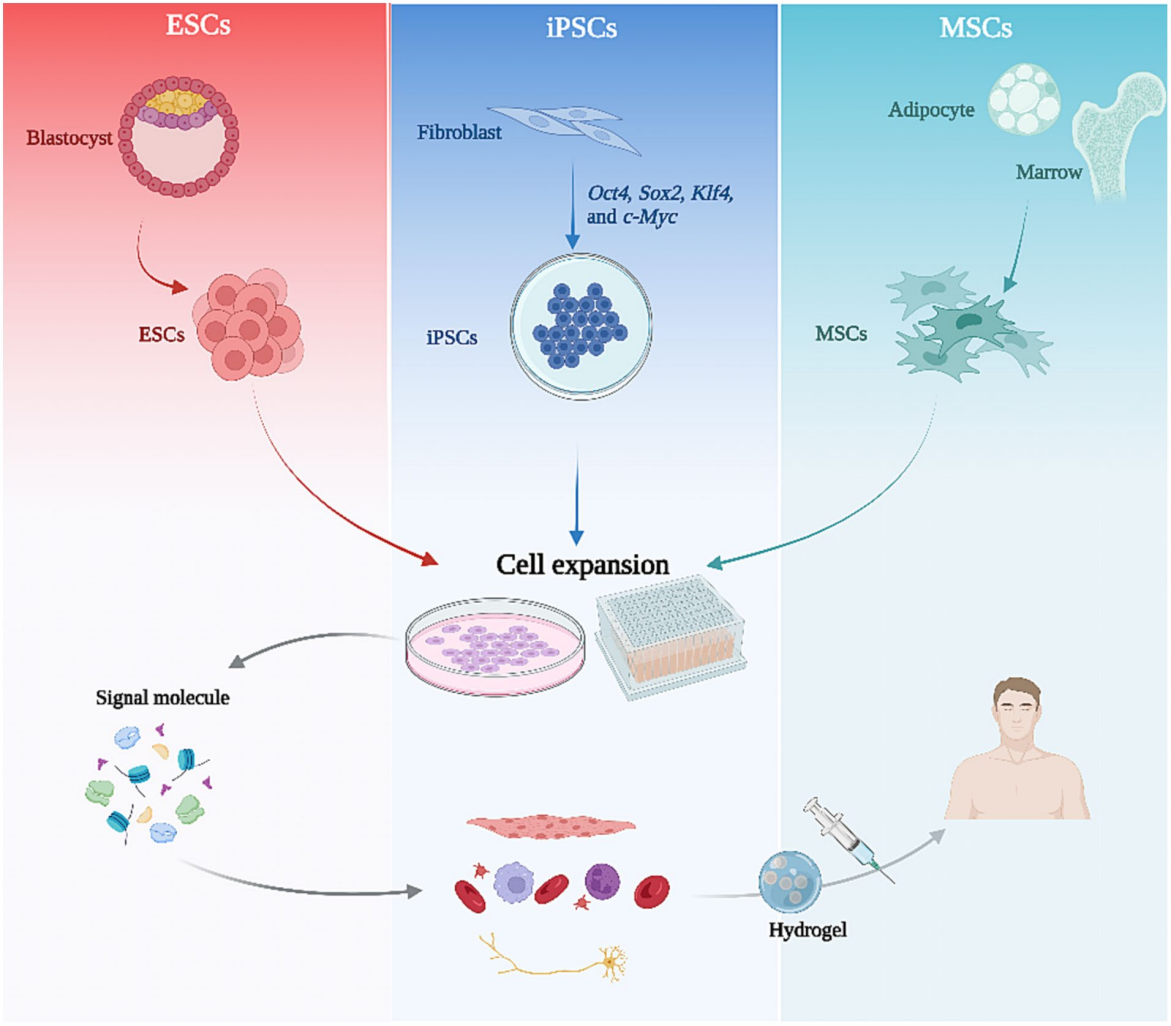
The flowchart of stem cell therapy. Various stem cell types are initially isolated from tissues and subsequently expanded through 2D or 3D culture. Following the regulation of culture conditions and signaling molecules, these stem cells can be directed towards specific differentiation pathways, ultimately resulting in tissue-specific cells that can be utilized for therapy via hydrogel encapsulation injection.

In order to maintain the multigenerational self-renewal capacity and differentiation ability of SCs, specialized culture systems are required to support this. Different biomaterials in the culture medium can impact the differentiation potential and the amplification capacity of SCs. Media containing oligopeptide-grafted hydrogels have been shown to enhance the proliferation and pluripotency of human ESCs and iPSCs (Chen et al., 2017). The use of culture systems containing human plasma and human embryo extracts maximizes the number of passages while maintaining the self-renewal and differentiation potential of iPSCs (Wang et al., 2012). In addition, compared to 2D cultures, 3D culture systems can better mimic the microenvironment of SCs *in vivo* and enhance the stemness of different SC species (Al Madhoun et al., 2016; Thakur et al., 2022). For example, a combination of 3D cell culture and natural brain tissue extracts can accelerate the differentiation of SCs into neuronal phenotypes (Azizi et al., 2018).

For SC therapy, the most crucial step is to direct the differentiation of SCs toward the target cell type by regulating culture conditions and signaling molecules. This can be achieved by mimicking the signaling pathways and microenvironment during embryonic development. Studies have shown that inner ear development is closely linked to fibroblast growth factor (FGF) signaling (Alvarez et al., 2003). Stimulating this pathway in human ESCs can induce two types of ear progenitor cells that differentiate into inner ear hair-like cells and auditory neurons, respectively (Chen et al., 2012). Mild activation of Wnt signaling promotes the differentiation of MSCs into chondrogenic cells (Schizas et al., 2021). The adhesion and growth characteristics of cells can also be influenced by culturing them on the surface of nanomaterial composites, which triggers mechanotransduction-induced changes in gene expression through changes in cytoskeletal structure. Mouse kidney-derived SCs have been induced to differentiate into podocytes or proximal tubular cells in this way (MacGregor-Ramiasa et al., 2017). In contrast, in neural differentiation of SCs, chemical inducers or growth factors, including retinoic acid (RA), brain-derived neurotrophic factor (BDNF), and nerve growth factor (NGF), are required (Gupta and Singh, 2022). Finally, the most direct way to induce directed differentiation of SCs is through transcription factor regulation. In addition to the above-mentioned transcription factors that can reprogram fibroblasts to iPSC, different transcription factors are required to induce differentiation of the required cell population (Ng et al., 2021). Overexpression of *NR5A1* and *RUNNX1* or *RUNX2* induces the differentiation of iPSC into human ovarian granulosa cells (Pierson Smela et al., 2023). The combined action of transcription factors, *GATA4*, *Tbx5*, *MEF2C*, and *Hand2,* reprograms mouse tail-tip and cardiac fibroblasts to cardiomyocyte-like cells with cardiac function *in vitro* (Song et al., 2012). Overexpression of *GFI1*, *Pou4f3*, and *ATOH1* directly induces the transformation of human fibroblasts into inner ear hair cell lineages (Duran Alonso et al., 2018).

Following combination and optimization of the above methods, directed differentiation of SCs can be achieved. After ensuring the validity and stability of the differentiation process, cell identification and functional validation, including cell phenotype analysis, gene expression analysis and functional assessment, are required to confirm that the differentiated cell types are as expected, thus ensuring that the resulting cells have the desired properties and functions.

Finally, differentiated and validated SCs are transplanted into patients via different vectors and scaffolds. At this stage, enhancing the retention of SCs in tissues is critical to the efficacy of the therapy. The most commonly used modality is injecting a saline suspension of the SCs directly into the target organ or tissue (Mousaei Ghasroldasht et al., 2022). However, due to the low adhesion of saline, only a small number of cells may remain in the tissue following injection. Therefore, a medium with higher adhesion properties is needed as a vehicle for SCs transplantation, such as a hydrogel (Nayagam et al., 2012; Niu et al., 2019). Nanohybrid hydrogels containing sufated glycosaminoglycan-based polyelectrolyte complex nanoparticles (PCN) are able to mimic extracellular matrices and contain a variety of bioactive factors to improve the implantation rate of neural SCs, while enabling cellular responses after central nervous system injury (Jian et al., 2018). Gelatin methacrylate (GelMA)/sodium alginate (Alg) (GelMA/Alg) hydrogels also contribute to the reduction of cellular damage after the implantation of neural SCs (Chen et al., 2023). Hydrogels of different compositions have played essential roles in cardiac infarction, skin regeneration, liver regeneration, etc., (Mardpour et al., 2019; Ke et al., 2020; Gong et al., 2022).

In summary, a complete SC therapeutic process comprises three significant aspects: SC generation and amplification, targeted differentiation and application, and selection of the optimal technical route to achieve regeneration and functional recovery of damaged tissues and organs is required for different clinical areas (Figure 2).

## Applications of SC therapy

Cell therapy for organ and tissue regeneration encompasses a range of methods aimed at repairing or regenerating damaged tissues or organs by introducing exogenous cells into the body. Stem cell therapies, among other approaches, constitute a significant aspect of this field, harnessing the regenerative potential of specific cell populations to restore tissue function in conditions ranging from degenerative diseases to injuries.

### Cell therapy for organ and tissue regeneration

Cell-based therapies operate through various mechanisms, encompassing cellular differentiation, secretion of bioactive molecules like growth factors and cytokines, modulation of immune responses, and facilitation of tissue repair and remodeling. Degenerative and injurious diseases, including circulatory, endocrine and neurological disorders, have the potential to be restored through SC therapy (Rossi and Cattaneo, 2002; Boyle et al., 2006). The first clinical applications were in the hematological sector, involving transplantation of hematopoietic stem cells (HSCs) from the blood system (Eaves, 2015). HSC transplants have now become the standard of care for hematological malignancies and hereditary blood cell disorders (Bordignon, 2006). Graft-versus-host disease (GVHD) can be minimized by analyzing genes within the human leukocyte antigen (HLA) region to find the best HLA-matched donor and recipient. To avoid the limitations of donor matching and potential immune complications, genetic correction or gene editing of patient’s own HSCs has dramatically improved the efficiency of transplantation therapy for hematological disorders (Morgan et al., 2017). Wiskott-Aldrich syndrome (WAS), characterized by macrothrombocytopenia, eczema, autoimmunity, and lymphoid malignancies, is caused by the expression of mutated forms of the *WAS* gene. This mutation has been corrected in the patient’s own HSCs by lentiviral transfection of the correct gene, followed by infusion of the modified HSCs into the patient, who showed improvement in immune function and clinical symptoms (Aiuti et al., 2013). In sickle cell disease, the hemoglobin abnormality is reversed by the introduction of the globin genes (γ-globin, γ/β-globin hybrids, and anti-sickle β-globin) into HSCs via γ-retroviral and lentiviral vectors or by directly targeting the fetal γ-globin suppressor gene *BCL11A* (White et al., 2023).

SC therapy has also shown strong potential in the treatment of deafness. Combined treatment of ESCs with insulin-like growth factor-1 (IGF), epidermal growth factor (EGF), and bFGF can induce ESCs to express markers of inner ear progenitor cells, including *ATOH1* (Li et al., 2003). After co-culture of ESCs/iPSCs and stromal cells from embryonic chicken egg sacs, Oshima et al. identified a class of hair bundle cells with short microvilli that have electrophysiological properties resembling immature hair cells (Oshima et al., 2010). This method further completes the progressive differentiation from SCs to hair cells. Treatment of hereditary hearing loss with SCs also requires the aid of gene editing. In deaf patients with *MYO7A mutation*, CRISPR/Cas9 gene correction in iPSCs is required to restore normal morphology and function of the differentiated hair cell-like cells (Tang et al., 2016).

Cell-based SCs therapies have also been gradually refined for the treatment of heart and skin diseases. Cardiomyocytes are fully differentiated cells and have a limited regenerative capacity that determines the irreversible loss of cardiac function after injury (Eschenhagen et al., 2017). The re-differentiation of cardiomyocytes from ESCs and iPSCs is expected to further improve the function of damaged cardiac tissues (Mummery et al., 2012). With the addition of gene editing, SCs have been used to improve the treatment of cardiac diseases, including the introduction of *Akt1* to enhance the proliferation of cardiac progenitor cells (Noiseux et al., 2006), and modification of the *SDF-1/CXCR4* genes to facilitate the recruitment of cardiac SCs (Zhang et al., 2008; Tang et al., 2009). In terms of skin wound healing, SCs treatment mainly reduces healing time, risk of wound contracture and scar formation (Nourian Dehkordi et al., 2019).

In summary, cell-based SCs therapies work by direct replacement of the damaged tissues with cells derived from differentiation of normal SCs to restore tissue function or by correcting the abnormal SCs with gene editing so that normal tissue and organ function can be restored. While demonstrating potential in facilitating tissue regeneration and potentially reversing specific pathological conditions, several challenges persist, encompassing immune rejection concerns, ethical considerations, and the risks associated with unregulated cellular behavior subsequent to transplantation.

### Cell-free paracrine therapy

There is growing evidence that in addition to direct cell replacement therapy, SCs, particularly MSCs, secrete proteins, growth factors, cytokines, and chemokines that exert influence on the surrounding cells or microenvironment to support tissue regeneration (Han et al., 2022). These secretory products, collectively known as the SCs secretome, are able to modulate the microenvironment of the damaged tissue by affecting the signaling pathways in different cell types, including tissue-specific cells, immune cells, vascular endothelial cells, and fibroblasts in the extracellular matrix (ECM) (Konala et al., 2016; Praveen Kumar et al., 2019; Daneshmandi et al., 2020). Due to their immunomodulatory properties, MSCs produce different regulatory factors to modulate the immune response in the microenvironment after homing and migrating to sites of injury (Volarevic et al., 2017). Immune checkpoint inhibitors, such as anti-PD-1/PD-L1, used for cancer therapy can also induce autoimmune type 1 diabetes, while MSCs-derived exosomes significantly prevent anti-PD-1/PD-L1-induced diabetes in mice (Kawada-Horitani et al., 2022). Bone marrow-derived MSCs were found to promote tendon-bone healing in the rotator cuff of rats by secreting TGF-β to regulate macrophage polarization via the Smad2/3 pathway. Inhibition of the M1 macrophage phenotype and promotion of the M2 phenotype was thought to contribute to tissue regeneration (Chen et al., 2021). In addition, human MuSCs, a type of muscle SCs, can secrete mediators such as heme oxygenase-1 and prostaglandin E2 to inhibit T lymphocyte proliferation, induce Treg-like cell production and suppress the cytotoxic response of CD8+ T lymphocytes (Charrier et al., 2022). Thus, SCs can produce various mediators to act on multiple immune cells, including macrophages and T cells, and play a pivotal role in regulating the immune microenvironment.

SCs can also secrete factors that promote angiogenesis in the microenvironment while they themselves differentiate into suitable cell types to replace the damaged cells (Xia et al., 2019). This is the case in fetal skin where the SC secretome promotes HUVEC cell proliferation and angiogenesis by enhancing the transcriptional activity of targeted genes associated with fetal skin regeneration and angiogenesis, including *VEGF*, *Ang-1*, *Ang-2*, and *PLGF* (Boyle et al., 2006). ESCs-derived MSCs can promote angiogenesis and nerve regeneration through paracrine secretion, thus, improving neurological deficits and reducing infarct volumes in ischemic rats (Asgari Taei et al., 2021). Further proteomic analysis revealed that the cysteine-rich protein Cyr61 (also known as CCN1) is a pro-angiogenic factor that mediates vascular endothelial cell migration and angiogenesis through integrins α_v_β_3_ and AMPK (Estrada et al., 2009; Park et al., 2015; Li Z et al., 2019). Similar to Cyr61, MSC-derived heparinases also promote angiogenesis via integrin pathways (Hu et al., 2015). The microenvironment also contains transport systems such as lymphatic vessels which can be regulated by SCs. When quiescent SCs shift to the activated phase, they can change the expression of angiopoietin-like protein 7 (*Angptl7*) to *Angptl4*. This results in a switch from promoting lymphatic drainage to promoting lymphatic dissociation and reducing drainage, thus mediating lymphatic remodeling (Gur-Cohen et al., 2019). In summary, the paracrine secretions from SCs can regulate lymphatic drainage and promote angiogenesis to ensure nutrient supply while replenishing damaged cells.

In addition to the normal microenvironment, SCs can also regulate the tumor microenvironment (TME). MicroRNA-100-rich exosomes derived from MSCs can inhibit the expression of VEGF in breast cancer cells through mTOR/HIF-1α signaling, ultimately inhibiting angiogenesis in the TME (Pakravan et al., 2017). However, in bladder cancer, the secretome of adipose-derived MSCs promotes the proliferation and invasion of cancer cells *in vitro* (Maj et al., 2018). Thus, regulation of the TME by the secretome of SCs can be bidirectional depending on the tissue involved. However, based on the secretory property of MSCs, therapeutic modalities that use MSCs as carriers for targeted delivery of treatment agents are now emerging (Hu et al., 2010). From delivery of cytokines such as IFN-β (Studeny et al., 2004) and IL-2/IL-12 (Gao et al., 2010; Bae et al., 2022) to regulate the immune microenvironment (CD8^+^ T cells, NK cells), to delivery of drugs such as paclitaxel (Pessina et al., 2013), doxorubicin (Zhao et al., 2017), and photoresponsive agents for photodynamic therapy or photothermal therapy (Ouyang et al., 2020), to today’s delivery of suicide genes such as TRAIL (Li M et al., 2019) and herpes simplex virus-thymidine kinase (*HSV-TK*) (Oraee-Yazdani et al., 2023), the use of MSCs as a therapeutic vector has been progressively refined.

In summary, cell-free therapies based on SCs have shown great promise with their ability to modulate the tissue microenvironment for the treatment of more diverse diseases than cell therapies in which SCs are re-differentiated to replenish damaged cells. Cell-free paracrine therapy offers several advantages over cell-based therapies, including reduced risk of immune rejection, simplified storage and administration processes, and potentially fewer safety concerns (Table 2).

**Table 2:** Applications of stem cell therapy.

	Cell therapy	Cell-free paracrine therapy
Treatment principle	The inherent ability of stem cells to undergo self-renewal and differentiation	The capacity of stem cells to secrete and generate various bioactive substances
The main acting substance	Stem cells	Stem cells secretome (proteins, exosomes, and active factors)
Therapy method	Replace damaged or abnormal cells	Regulate the microenvironment
Clinical application	Hematopoietic stem cells transplantation; regeneration of inner hair cells, cardiomyocyte, and hypodermal cell	Suppressing autoimmunity in type 1 diabetes; promoting angiogenesis of skin and brain tissue; regulating the tumor microenvironment
References	Oshima et al. (2010), Eaves (2015), and Nourian Dehkordi et al. (2019)	Boyle et al. (2006), Pakravan et al. (2017), Maj et al. (2018), Asgari Taei et al. (2021), and Kawada-Horitani et al. (2022)

## Current challenges

In the current phase of rapid development in SC-based therapies, it is still important not to overlook some of the problems they pose. The first issue to be considered is the source of SCs as there are ethical and legal considerations (King and Perrin, 2014). The use of ESCs is subjected to ethical debates and legal limits, while the acquisition and amplification of adult SCs are technically tricky and have quality control issues (Chen et al., 2020). Another issue arising from prolonged continuous culture is the loss of cell viability, leading to reduced proliferative and differentiation capabilities. Addressing this necessitates the use of new materials, such as silica nanoparticles, for the long-term preservation of stem cells in a desiccated state (Gallina et al., 2015). Secondly, the efficiency and direction of differentiation of SCs is a major issue as this determines the effectiveness of the treatment. Directed differentiation is a complex process that we do not yet fully understand and many factors, such as cell culture conditions, cytokines, and signaling pathways can influence the process (Kim et al., 2016). Therefore, more research is still required to better control the direction and quality of differentiation of SCs to prevent adverse events such as tumorigenesis (Andrews et al., 2022). Another crucial determinant of stem cell therapy is the capacity to target cellular migration. Prior to assuming their role in differentiation, stem cells must be effectively delivered to the intended site. Currently, most stem cell therapeutic approaches employ intravenous drug delivery, which exhibits limited efficacy in facilitating targeted migration from blood circulation to tissues (Liu et al., 2020). Survival of the transplanted SCs is another major issue facing SC therapy. SC therapy, characterized by its low expression of MHC and HLA, holds the potential to achieve reduced immunogenicity and significantly enhance the suppression of the graft-versus-host response. However, owing to the limitations in pre-expansion technology associated with SC therapy, its immune privilege is progressively compromised. Upon infusion into the human body, the presence of inflammatory factors within the body further escalates the immunogenicity of SCs, thereby elevating the risk of rejection (Barrachina et al., 2017). Cell survival and growth after transplantation are influenced by the host immune system, since the host immune responses to the allogeneic cells directly contributes to graft rejection (Sanz-Ruiz and Fernández-Avilés, 2018). A possible solution for allo-rejection is to knockout immune-related genes by gene editing to generate immune-compatible SCs (Ye et al., 2020). Further research will be required to resolve these and other challenges to successfully translate SCs therapies to the clinics.

## Summary and perspectives

Since Ernst Haeckel first identified SCs in 1868, the development of these cells had gone through several critical stages. Initially, SCs were isolated and identified from various tissues, followed by the development of iPSCs and the combination of gene editing with SCs, leading to the progressive refinement of SC therapy. The most direct application for SCs is cell-based therapy, owing to their multi-directional differentiation capabilities. This approach involves the injection of SCs, both allogeneic and genetically modified autologous SCs, into the sites of disease or injury to promote tissue regeneration and functional recovery. The administration of cardiopoietic stem cell injection, induced by a cardiogenic growth factor, effectively enhanced cardiac function in patients with chronic heart failure during a clinical trial. Notably, no adverse effects on the heart or systemic toxicity were observed among the subjects (Bartunek et al., 2013). The deficiency of arylsulfatase A (ARSA), an inherited disorder known as metachromatic leukodystrophy (MLD), can be addressed through *in vitro* lentiviral transduction of autologous hematopoietic stem cells with cDNA encoding ARSA. This approach leads to enhanced ARSA activity and reduced brain damage (Fumagalli et al., 2022). An alternative application is cell-free therapy, utilizing the secretory ability of SCs, is also a critical approach. SC secreted factors can modulate the target tissue cells and the microenvironment, including the immune microenvironment and angiogenesis. Allogeneic expanded adipose-derived mesenchymal stem cells (Cx601) have been proven to secrete immunomodulators and anti-inflammatory factors, and have certain potential in the treatment of inflammatory bowel disease, especially in the treatment of anal fistula in patients with Crohn’s disease (Panés et al., 2018). The latest therapeutic approach is to use SCs as vehicles for the targeted delivery of effectors, drugs, and genes into damaged tissues or tumors to exert the appropriate regulatory effects. The potential of oncolytic adenovirus as an antitumor therapy is limited in central nervous system tumors due to the presence of the blood–brain barrier. However, a clinical trial demonstrated that delivery via neural stem cells (NSC) facilitated safe and efficient transportation of oncolytic adenovirus to the tumor site (Fares et al., 2021).

However, SC therapy also faces a number of safety issues. Allogeneic SCs can trigger the patient’s immune system, leading to graft rejection, while excessive proliferation and differentiation of transplanted SCs may lead to tumor formation. Ensuring the safety of SC therapy is, therefore, a significant challenge. It is also crucial in SC therapy to ensure that SCs can differentiate directionally into target cell types and maintain their function and stability. Further research and improved differentiation techniques are needed to ensure that differentiated cells have the desired characteristics.

In conclusion, as an essential therapeutic tool in regenerative medicine, SC therapy plays a vital role in a number of ways, both in the cells themselves and in their secreted components. With a better understanding of the properties and functions of SCs, it is expected that more diseases and injuries will be able to benefit from SC therapy.

## Author contributions

JW: Writing – original draft. GD: Writing – original draft. SW: Project administration, Supervision, Writing – review & editing. SL: Data curation, Investigation, Writing – review & editing. PS: Formal analysis, Investigation, Writing – review & editing. KL: Data curation, Formal analysis, Writing – review & editing. XX: Funding acquisition, Project administration, Writing – review & editing. ZH: Conceptualization, Funding acquisition, Writing – review & editing.
